# Impact of Regular Breathing Exercises on Blood Pressure Phenotypes and BMI in Young Male Individuals: A Narrative Review

**DOI:** 10.7759/cureus.90027

**Published:** 2025-08-13

**Authors:** Amrit Podder, Sariya Nazim, Ashwani Sharma, Angshuman De, Vishal Singh, Jayballabh Kumar, Devesh Kumar, Challa Sri Varsha, Parth Jani

**Affiliations:** 1 Department of Physiology, Teerthanker Mahaveer Medical College and Research Centre, Moradabad, IND; 2 Department of Biochemistry, Ramakrishna Mission Seva Pratishthan (Sishumangal Hospital), Kolkata, IND; 3 Biochemistry and Quality Assurance, Apollo Clinic (FMC Healthcare), Kolkata, IND; 4 Biochemistry, Welkin Medicare, Kolkata, IND; 5 Physical Education and Sports, Pondicherry University, Pondicherry, IND; 6 Department of Physiology, Autonomous State Medical College, Shahjahanpur, IND; 7 Department of Medicine, Saveetha Medical College and Hospital, Chennai, IND; 8 Department of General Medicine, All India Institute of Medical Sciences, Rajkot, IND

**Keywords:** blood pressure, bmi, hypertension, regular breathing exercise, young individuals

## Abstract

Pranayama is the practice of manipulating breathing, which serves as a dynamic link between the mind and body, and is well known in traditional Indian medicine. When combined with breath control, pranayama alters the heart’s output and the blood flow to the kidneys and liver, which in turn controls blood pressure (BP). Vascular abnormalities change the total peripheral resistance, which makes the arteries more rigid and contributes to the development of hypertension. Autonomic modulation is altered by yoga breathing patterns, which results in modifications to cardiovascular functioning that are sustained and continuous, as well as central and autonomic processes, i.e., mechanical and hemodynamic. This narrative review was conducted using sources from PubMed, Scopus, MEDLINE, and Web of Science, in which we included published literature starting from 1990. We reviewed the existing literature on regular breathing exercises and their effect on blood pressure and body mass index (BMI). In this review, we narrated the influence of regular breathing exercises on blood pressure and BMI with possible mechanistic pathways, such as the roles of hemodynamic fluctuation, endothelial dysfunction modulation, vascular stiffness, and cardiac remodelling, to provide conclusive and comprehensive understanding regarding their intricacy among young individuals.

## Introduction and background

Featuring traditions in yoga and meditation, breathing practices are growing more and more prominent among scientists as non-pharmacological ways for improving physical and mental health. Methods such as "alternate nostril breathing" and "diaphragmatic breathing" have been associated with enhanced autonomic control, decreased stress levels, and improved cognitive function [1,2]. Additional "pranayama" forms include abdominal breathing, vocalized or chanting breathing, forceful breathing, and nostril breathing (double, single, or alternating), all of which are performed at various intensities and speeds [3]. Yoga and pranayama acquired popularity in the middle of the 20th century after being introduced to the West in the late 18th century. Since then, as fascination with wellness and alternative treatment approaches has developed, breathing techniques have expanded in appreciation. In individuals with essential hypertension (HTN), the technique of 2:1 breathing may be beneficial since it lowers blood pressure immediately and lowers stress levels in the body [4]. The objective of this narrative review is to develop a comprehensive understanding regarding the effect of regular breathing exercises on blood pressure and body mass index (BMI) in young individuals [5]. In this review, we narrated the influence of regular breathing exercises on blood pressure and BMI with the possible mechanistic pathways, such as the roles of hemodynamic fluctuation, endothelial dysfunction modulation, vascular stiffness, and cardiac remodelling, to provide conclusive and comprehensive understanding regarding their intricacy among young individuals.

## Review

Methodology

This narrative review was conducted by systematically evaluating literature on the impact of regular breathing exercises on blood pressure phenotypes and BMI in young male individuals. Articles published in English were identified through PubMed, Scopus, and Google Scholar. Inclusion criteria were original research (randomized controlled trials, quasi-experimental, and observational studies) involving healthy males aged 18-30 years, with breathing exercises as the primary intervention and reporting blood pressure and/or BMI outcomes. Exclusion criteria included studies with female or mixed-gender participants without separate male data, participants with chronic illnesses, interventions combined with other modalities, and non-full-text or non-English publications. The review adhered to the SANRA (Scale for the Assessment of Narrative Review Articles) guidelines to ensure methodological rigor.

Search Strategy

Searches were run in the already mentioned database using combinations of keywords joined with OR within concept groups and AND between concept groups (e.g., (breathing exercise OR pranayama) AND (hypertension OR blood pressure) AND (BMI OR "body mass index") AND (young OR "young adult")); searches were last updated on June 30, 2025.

Definitions

The definitions of regular breathing exercises and hypertension are mentioned in Table 1.

**Table 1:** Definitions

Term	Definition
Regular breathing exercise	Performing at least 15 minutes each day for at least five days a week was considered as regular breathing [5]
Hypertension	Hypertension (HTN) is defined as a systolic blood pressure (SBP) of 130 mmHg and a diastolic blood pressure (DBP) of more than 80 mmHg [5]
2:1 breathing	Exhalation is twice inhalation [4]

Epidemiology

According to data from various studies, less than 10% of all deaths worldwide were attributable to cardiovascular diseases (CVDs) in the previous century, but during the past 20 years, we have also seen a sharp increase in these numbers to 30% in countries with lower socioeconomic status, while the global total mortality burden for the same condition still stands at 80% [6]. Mathers et al. in 2001 investigated and came to the conclusion that cardiovascular illnesses account for around 28% of deaths in middle- and low-income countries, and more than 50% of all deaths in high-income countries [7]. The study conducted by Murray and Lopez in 1996 stated that CVDs will be the major cause of mortality worldwide by 2020, mostly due to its rise in low- and middle-income nations. Similar to developed countries since the mid-20th century, CVDs became the main cause of death in the underdeveloped world by 2001 [6].

Physiology of pranayama

System of Respiratory Organs' Breathing Biomechanics

“Tidal breathing” is the term used to describe normal breathing, which is accomplished by a group of respiration muscles, which are sometimes also known as the “respiratory pump” [8]. The diaphragm, i.e., the primary breathing muscle, expands and contracts when inspiration begins normally, causing the abdomen to expand and the lower ribs to move upward and outward [9]. This transdiaphragmatic pressure is responsible for the creation of the gradient of the transpulmonary pressure [9], but as the amount of breathing rises, the expiratory muscles, which comprise the abdominal muscles that, when engaged, draw the abdominal wall inward, causing the diaphragm to rise superiorly inside the chest cavity and compress the actively engaged lungs [10]. These physiological actions by the respiratory muscles in a systematic manner benefit the body functioning by optimizing the homeostatic mechanisms and may improve cardiovascular function by promoting optimal blood pressure regulation and supporting vascular health [8,9,10]. 

Pranayama Cycle

"Full inhalation," "holding off after inhaling," "controlled exhalation," and "breath holding" are the four different breathing methods used in pranayama. "Full inhalation" focuses on quiet, and rhythmic breathing while concentrating on a steady, intentional inhaling of oxygen. The body and mind are energized by this method. For beginners, "holding off after inhaling" is a short pause that enables them to hold their breath without exertion or movement. This phase encourages serenity and concentration. The "controlled exhalation" focuses on gradually increasing the breath, which calms the mind and promotes relaxation and mental clarity. "Breath holding" helps the practitioner feel calm and satisfied on the inside. More experienced practitioners’ respiratory systems and lung capacities are strengthened by their ability to hold their breath for prolonged periods of time. These are some of the most commonly practiced breathing exercises in traditional practice [11].

Exchange and Ventilation of Gases

The degree of diaphragm movement and changes in lung capacities have been shown to have a significantly positively correlated in peer-reviewed studies, suggesting that diaphragmatic breathing facilitates slow breathing [12]. According to the study's findings, the diaphragm muscle's appropriate compression and relaxation aid in controlling both frictionless breathing and the pressure within the abdominal cavity [13]. When compared to normal breathing patterns or controlled breathing at 15 respirations per minute, it is noticeable that in healthy individuals, controlled slow breathing at six respirations per minute reduces the reaction of chemoreflex to hypoxia and hypercapnia [14]. The maintenance of oxygen, carbon dioxide, and pH levels of blood in an equilibrium requires exact control of lung breathing’s biomechanics. If the respiratory rate decreases, the tidal volume must be increased to maintain the minute ventilation estimated through multiplying the respiratory rate by the tidal volume unaffected [15], provided that an increase in ventilation reliability becomes independent of increasing respiratory rate as it increases physiological dead space, which consists of the total of anatomical dead space and alveolar dead space [16]. It has been demonstrated, on the contrary, that lowering respiratory rate and raising tidal volume enhance ventilation efficiency by promoting alveolar recruitment and distension [17]. A study has confirmed this by determining the saturation of the arterial oxygen during spontaneous breathing and respiration at different time intervals during relaxation and during exercise [18].

Hemodynamic Fluctuations

Normal inspiration causes a reduction in intrathoracic or intrapleural pressure that is transported to the right atrial chamber of the heart, which increases the pressure slope between the systemic circulation and the right side of the heart, so filling the right atrial chamber, increasing the right ventricular stroke volume, and increasing the venous return to the heart [19]. It has been shown that slow breathing, up to six breaths per minute, increases venous return [20]. The diaphragm additionally improves diaphragmatic breathing performance by simplifying the passage of the inferior vena cava (IVC) and aorta [21]. Although studies on diaphragmatic breathers show increased venous return efficiency, particularly with slow, deep breathing, diaphragmatic descent transiently alters intra-abdominal and intrathoracic pressures and can allow partial collapse of the inferior vena cava (IVC) during inspiration. This IVC collapse is commonly a normal, physiological finding reflecting normal respiratory variation. However, when the collapse is pronounced or occurs together with hypotension, tachycardia, poor skin perfusion, or other clinical findings, it may reflect low intravascular volume or compromised venous return (e.g., hypovolemia or obstructive processes). Thus, the presence of inspiratory IVC collapse should be interpreted in the clinical context rather than as an isolated marker of benefit or harm. [22]. Another study showed that when respiration is slowed, the association between vasomotion and breathing leads to oscillations in capillary blood flow. It was hypothesized that slow respiration might entrain while improving vasomotion, particularly whenever there is space for better oxygenation of the blood [23].

Blood Pressure and Heart Rate

Blood pressure and heart rate fluctuate as a result of these breathing adjustments in the cardiac output, stroke volume, and venous flow, as well as peripheral circulation [24]. Although changes in the cardiovascular system may affect respiration, it has been shown that breathing has a greater effect on the cardiovascular system [25]. In healthy individuals, acute exposure to regulated slow breathing (six breaths per minute) during short-term laboratory assessments has been observed to transiently increase heart rate and blood pressure compared to normal respiration rates. However, chronic practice of slow breathing over weeks to months has been shown to lower resting blood pressure, highlighting a difference between immediate physiological responses and long-term adaptations. [26]. Some hypothesize that there is a buffering of hemodynamic oscillations linked to the respiratory system since the pulsing blood flow is timed with the heartbeat [27]. On the other hand, a number of research revealed that controlled slow respiration substantially lowers mean blood pressure, which lends credence to this theory [28]. Cardiorespiratory coupling refers to the connections between blood pressure, heart rate, and breathing [29]. A study conducted by Pal GK et al. in 2004 evident that three months of regular breathing exercises enhanced the vagal activity, which caused a substantial decrease in the basal heart rate [30]. Bhavnanai AB et al. in 2011 concluded their study in the same direction by showing that breathing exercises may decrease blood pressure and heart rate by enhancing vagal activity through performing a significant reduction in sympathetic activity and sensitivity to the baroreflex mechanism [31]. In 2009, Pramanik T et al. noticed in their study that regular breathing exercises reduce the diastolic and systolic blood pressure by causing a reduction in total peripheral resistance [32].

Vascular Physiology

The hardening of arteries, which is usually associated with a variety of arterial problems, is caused by changes in an extensive number of genes that regulate vascular development, especially arterial growth and health [33]. Podder A et al. (2023) in their research came to the conclusion that changes in multiple anthropometric characteristics, including an individual's waist circumference and body mass index, are linked to elevated blood pressure when they found a positive correlation between blood pressure and obesity. Vascular integrity deterioration causes hypertension and other heart-related disorders. In individuals suffering from hypertension, a high arterial stiffness index affects the microvascular environment, causing problems with the homeostasis of the heart, brain, and nephrovascular system. These problems may lead to stroke, vertebral, carotid, and cerebral stenosis. Various studies also reported that the different forms of breathing exercises not only optimize the vascular physiology but also delay the pathogenic ageing process of the vessels [34].

Heart Rate Variability (HRV)

Heart rate variability (HRV) is the fluctuation in the interval between heartbeats, which measures the heart’s capacity to regulate itself and respond to stress. Although low HRV is a sign of compromised autonomic function and has been linked to an elevated risk of cardiac complications, higher HRV is associated with better cardiovascular health [35]. It has been shown that regular breathing exercises raise HRV via activating the vagus nerve, which is an essential part of the parasympathetic nervous system. A higher HRV is indicative of improved autonomic balance, which helps the heart better control its rhythm and adjust to stress. This might lessen the adverse effects of cardiac remodeling by enhancing the heart’s response to both internal and external stimuli [36]. According to Herawati I et al. (2023), breathing exercises may be used to manage hypertension without the use of medications. The results of breathing exercises, a non-pharmacological therapy for hypertension, were determined in this research. Nearly all of the included research shows that this effective strategy produces favorable results. This discovery may serve as the basis for the breathing techniques professionals use when treating patients with hypertension [37].

Role of Endothelial Dysfunction Modulation and Inflammation

A physiological vascular architecture is maintained through the maintenance of a proper optimal endothelial function. Degradation of nitric oxide (NO) happens when there is reactive oxygen species generation in excessive amounts, which facilitates the pathogenic vicious cycle necessary for dysfunction of the endothelial layer of the vessels. As it is evident that NO is a potent vasodilator, degradation of it accelerates the pathogenic vicious cycle, facilitating oxidative stress, which in turn results in less vasodilation. Apart from ROS, pro-inflammatory substances such as tumor necrosis factor α (TNF-α) act as precipitating factors for the development of dysfunction of the endothelium. Regular breathing exercises help in the maintenance of normal vascular architecture as there is sufficient synthesis of NO via stimulation of endothelial nitric oxide synthase (eNOS). This also suppresses the NF-κB signaling pathway which is a key regulator of genes involved in inflammation and thereby reducing vascular inflammation and limiting pro-inflammatory activity [38].

Role of Oxygen-Sensing Proteins in the Modulation of Vascular Architecture

**Figure 1:**
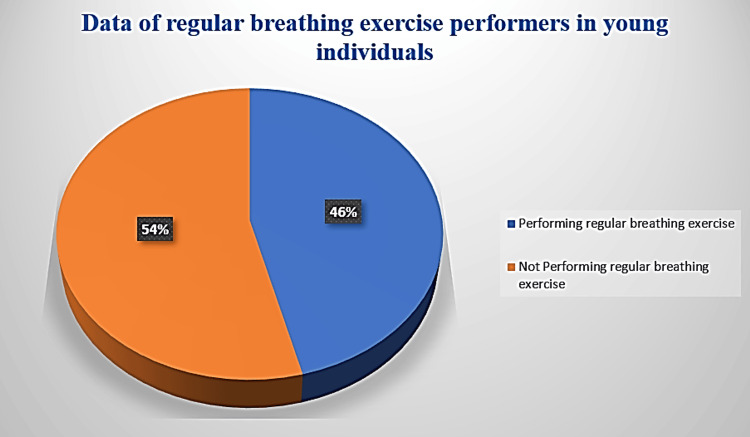
Average percentage of young male individuals performing breathing exercises. Source: [5]

The integrity of vessels degenerates due to increased lateral pressure delivered to the vascular wall. One of the commonly cited mechanisms for this pathogenic process is non-optimal levels of oxygen-sensing proteins. There is a significant reduction in the supply of oxygen to the tissues in vasoconstriction-induced hypertension due to an increased level of erythropoietin (EPO), an oxygen-sensing protein, which may alter the process of angiogenesis if not kept within normal limits. The other reason for the development of hypertension due to non-optimal EPO levels is the stated effect of its vasoconstrictor on tiny-resistance vessels, which provides an inverse effect over the acetylcholine-induced vasodilatory mechanism. Some studies suggest that elevated EPO may contribute to hypertension by increasing calcium ion influx into vascular smooth muscle cells via L-type calcium channels. The pathophysiology of arterial smooth muscle is regulated by vascular endothelial growth factor (VEGF), an oxygen-sensing protein, whose production is dependent on the hypoxia signaling pathway. Studies have been conducted to show that inhibition of VEGF leads to a rise in BP as it reduces the synthesis of nitric oxide. Breathing exercises help in breaking the vicious cycle responsible for the progressive degeneration of vascular architecture by modulating the pathway for the synthesis of nitric oxide and thereby keeping the oxygen-sensing protein within the optimal limit [39]. Nazim S et al. also found in their research that the proportion of young male individuals who regularly perform any form of the regular breathing exercise techniques are almost 46%. The diagramatic representation of their observation is shown in Figure 1, which they have depicted in tabular form [5]. Studies have also found that obesity is one of the major risk factors for intracranial hemorrhage, and since regular breathing exercises can contribute to weight management and improved cardiovascular health, they may indirectly help reduce this risk [40] and long standing hypertension can lead to aortic rupture [41]. It has been observed that patients having hypertension leads to rare entity saccular aneurysm with dissection and rupture in descending thoracic aorta [42]. The role of breathing exercises in enhancing the well-being in elderly individuals are already shown in some studies [43]. The similar mechanisms can be helpful evn in young individuals [5].

## Conclusions

Our review suggests that regular breathing exercises are linked to an improvement in young individuals' blood pressure. It is also associated with lowering the obesity indices. Although we found that the percentage of young individuals who are performing breathing exercises regularly is low, it may be recommended that young people should engage more in regular breathing exercises to help them manage with issues associated with high blood pressure. However, further research is required to determine if breathing exercises have an impact on blood pressure. Such techniques can potentially be incorporated into regular activities in educational, athletic, or professional settings, given the elevated level of stress and performance pressure in this population. We propose more community-focused observational studies to be conducted in this direction. Randomized controlled trials are also warranted to establish causality in this direction.
